# *Stellaria media* (L.) Vill.- A plant with immense therapeutic potentials: phytochemistry and pharmacology

**DOI:** 10.1016/j.heliyon.2020.e04150

**Published:** 2020-06-07

**Authors:** Oluwole Solomon Oladeji, Abel Kolawole Oyebamiji

**Affiliations:** a Natural Products Research Unit, Department of Physical Sciences, Industrial Chemistry Programme, College of Pure and Applied Sciences, Landmark University, PMB 1001, Omu-Aran, Nigeria; b Department of Basic Sciences, Faculty of Sciences, Adeleke University, Ede, Nigeria; c Department of Pure and Applied Chemistry, Ladoke Akintola University of Technology, Ogbomoso, Nigeria

**Keywords:** *Stellaria media*, Caryophyllaceae, Ethnopharmacological, Phytochemistry, Biological activities, Plant biology, Pharmaceutical science, Biochemistry, Pharmacology, Evidence-based medicine

## Abstract

*Stellaria media* Vill. is a representative of Caryophyllaceae family. The plant is widely dispersed all over the world and has been used as therapeutic substance since time immemorial. This review is aimed at exploring the chemical constituents and pharmacological activities of *S. media*. The findings revealed important secondary metabolites such as flavonoid, oligosaccharide stellariose, anthraquinone derivatives, fatty acid, steroid saponins and phenolic compounds. These bioactive metabolites displayed diverse pharmacological activities such as anti-obesity, antifungal, antibacterial, antioxidant, anti-proliferative, anti-inflammatory, analgesic, antidiabetic and anxiolytic activities. All findings revealed that *S. media* is a major species of Caryophyllaceae family. However, bioactive constituents and pharmacological potential of are not well appraised. Hence, extracts with established pharmacological activities should be subjected to bioassay guided isolation so as to obtain compounds with novel structural moieties prior to toxicogenetic appraisals.

## 1 Introduction

Medicinal herbs are regarded as the fundamental and safest therapeutic approach since primordial time and have significantly played life-saving roles in primary health care development [1, 2, 3]. According to World Health Organisation, herbal medicine is appraised as alternative therapeutic system to achieve total health care against various diseases such as gonorrhoea, syphilis, typhoid, malaria, cholera, measles, and ulcer [4]. Medicinal plants are natural resources, generally acceptable and assumed to have fewer side effects [5, 6]. Medicinal plants are widely distributed all over the world and their curative properties have been appraised on several ailments [7, 8]. *Stellaria media* Linn. is a perennial plant widely dispersed in cold and temperate regions [9]. Due to its geographical distribution, it has numerous common names, however, universally known as chickweed and about 120 species have been reported [10]. Different parts of the plant have been used to treat various gastrointestinal disorders, asthma, diarrhoea, measles, jaundice, renal, digestive, reproductive and respiratory tracts inflammations. They also lessen swelling and used as plasters for broken bones [11].

The appraisal of bioactive metabolites from different parts of *S. media* revealed over 80 secondary metaboltes. Some of these compounds are saponins, alkaloids, cardiac glycosides, fatty acids, tannins and terpenoids [12, 13, 14]. The crude extracts and isolated compounds of *S. media* demonstrated significant pharmacological activities such as anti-hepatoma [15], anti-obesity [16, 17], anticancer [18], antipyretic, anti-inflammatory [19], anti-oxidant [20], antimicrobial [21, 22] and anxiolytic potentials [23]. The plant has played remarkable drug discovery roles in conventional and modern medicine. However, there is no comprehensive information on the phytochemical and pharmacological activities of crude extracts and isolated compounds. Therefore, this review is tailored towards exploring the botanical description, traditional significance, phytochemical profile and pharmacological activities of the *S. media.*

## 2 Review methodology

Literature were explored from major scientific catalogues such as Science Direct, PubMed, MedLine, Google Scholar and Scopus with the keyword “*Stellaria media* L.,” “phytochemicals,” “ethnopharmacology” and “pharmacological activities”. Several published articles (2005–2019) were queried to procure information on phytochemistry and pharmacological assessments of *S. media.*

### 2.1 Ethnobotanical description

**Figure 1:**
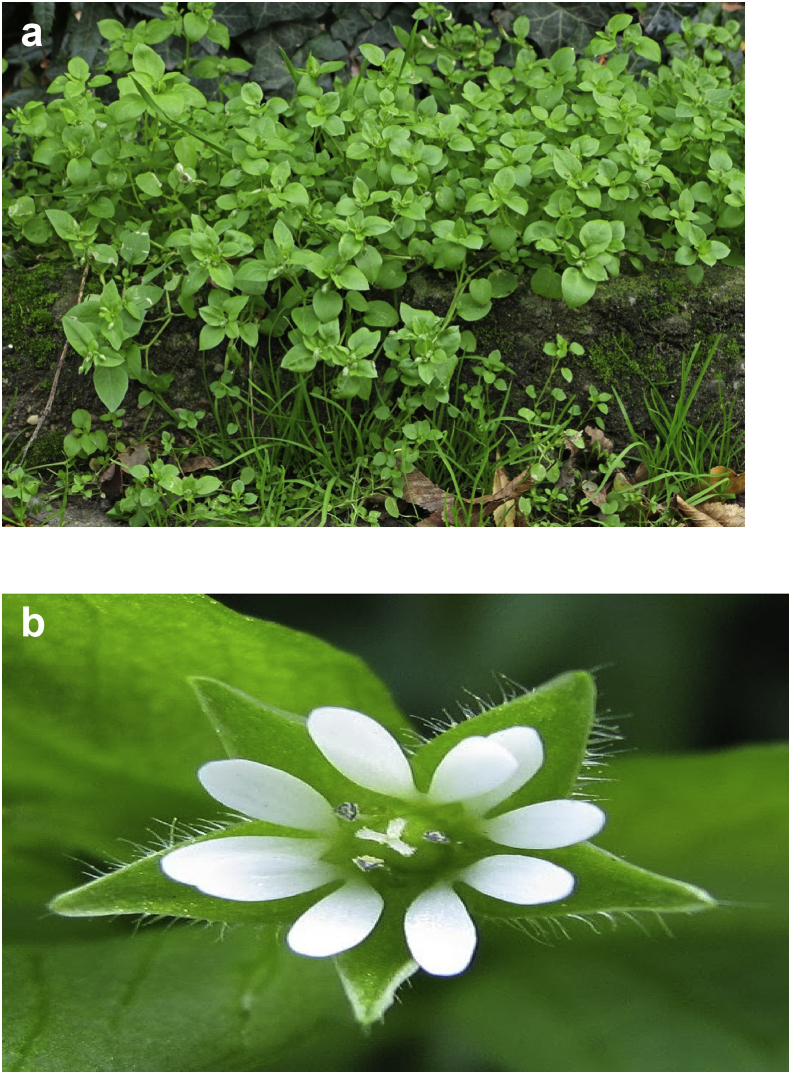
Images of different parts of *Stellaria media* ([a] whole plant [b] aerial part (flower).

*Stellaria media* L. germinates in autumn and springs flowers between May and October [24]. It has characteristic weak willowy stems, oval leaves, small white flowers with deep lobed petals followed quickly by the seed pods. The plant grows up to 40 cm in moist-fertile and nitrogen-rich soils especially in meadow, lawns and dumping sites (Figure 1). It is widely distributed mostly in Asia, North America, Africa and Europe, having distinctive fine hairs on one facet of the stem [25].

### 2.2 Taxonomy

The plant belongs to family Caryophyllaceae (Table 1).

**Table 1:** Taxonomy of *Stellaria media* L.

Kingdom:	Plantae
Subkingdom:	Viridiplantae
Division:	Tracheophyta;
Subdivision:	Spermatophytina;
Class:	Magnoliopsida;
Superorder:	Caryophyllanae;
Order:	Caryophyllales;
Family:	Caryophyllaceae;
Genus:	*Stellaria* L.
Species:	*Stellaria media* (L.).

### 2.3 Ethnopharmacology

Traditionally, leaves decoction of *S. media* has been reported for its curative applications. In Asia and tropical Africa, leaves decoction is used to treat acute gastrointestinal and respiratory diseases. Also, dried leaves are processed into pills, powders, or decoctions majorly to treat dermal infections, leg swelling, heart infections, thyrotoxicosis and haemorrhoids [16, 26]. In India, leaf decoction is used to dressed deep wounds, stop bleeding and lessen tumour [27], pulverized leaves, stem and root is used in form of plaster for dislocated bones and swelling [28]. The whole plant is used to treat asthma, bronchitis, pulmonary diseases and obesity [29]. The plant decoction has unique moisturizing and soothing properties which encourage its usage to relieve dermal itching, menstrual pain and mange [30]. Also, mixture of leaves, stem, flowers and root are often macerated and is effective in regulating psychological disorder, respiratory and reproductive tracts inflammations [31].

### 2.4 Phytochemical profile of *Stellaria media*

**Figure 2:**
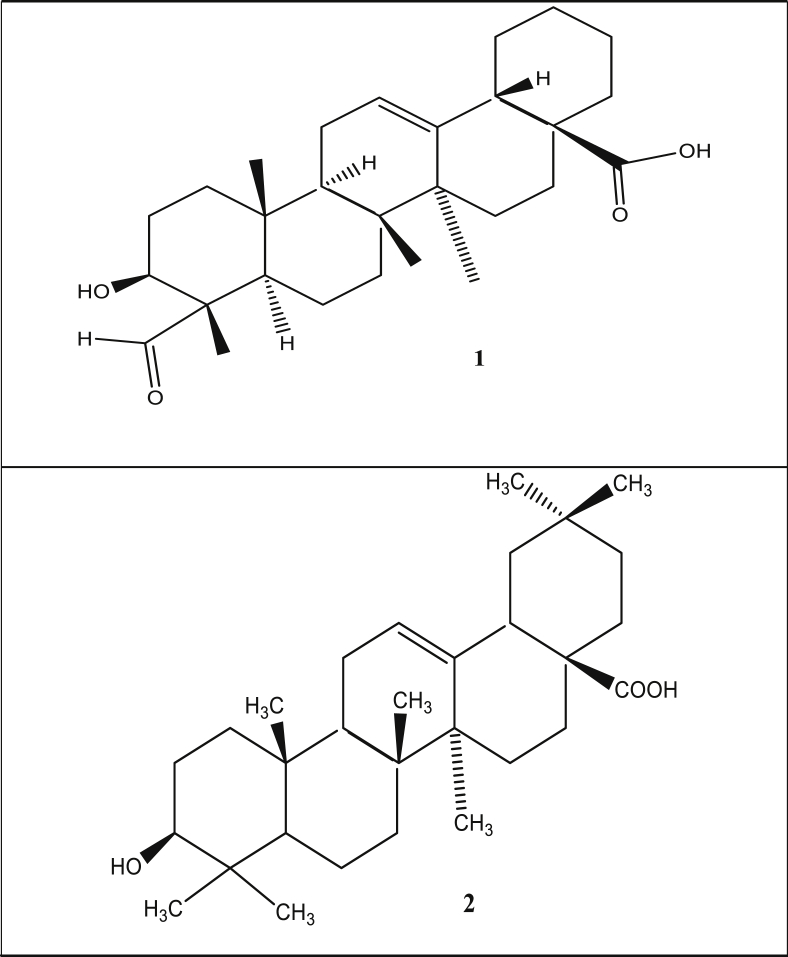
Triterpenoid saponins isolated from the *S. media* (1):- Gypsogenin, (2):- Oleanolic acid.

**Figure 3:**
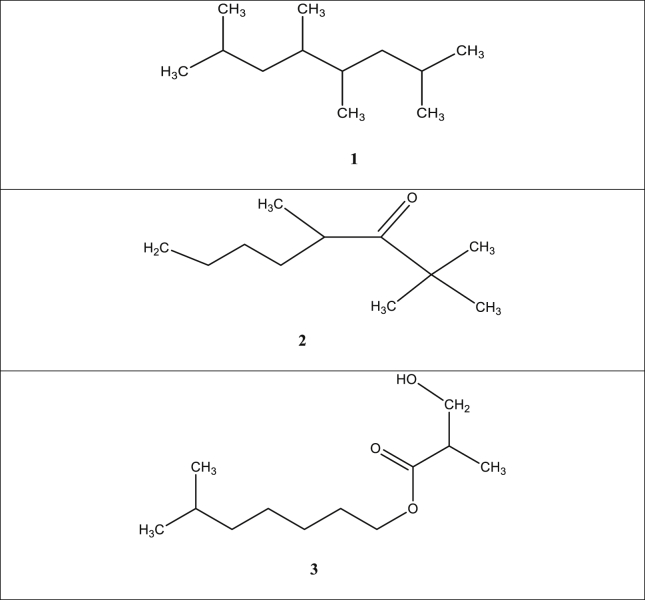
The chemical constituents isolated from aerial parts of *Stellaria media* Linn. **(1)** - 2, 4, 5, 7-tetramethyloctane, **(2)** - 2,2,4-trimethyloctan-3-one, **(3)** - 6-methyl heptyl-3′-hydroxy-2′-methylpropanoate.

Phytochemical appraisals of extracts and fractions of different parts of *S. media* have led to identification of about 50 bioactive metabolites. Few chemical compounds have been reported from bioassay isolation of different crude or purified fractions. Most of the bioactive metabolites reported till date belongs to phenolic compounds and saponins. Preliminary phytochemical investigation of ethanolic, ethyl acetate, methanolic, chloroform and aqueous extracts revealed the presence of alkaloids, saponins, cardiac glycosides, fatty acids, tannins, terpenoids and phenolic compounds, however, phlobatannins and anthraquinones were undetected [12, 14, 32]. Gas chromatographic-mass spectrometry (GC-MS) appraisals of purified fraction of leaf extracts revealed important bioactive metabolites such as luteolin, flavones, C-glycosyl flavones, vicenin-2, isoquercetin, genistein and apigenin [33]. In another study, vanillic acid, caffeic acid, *p-*hydroxybenzoic acid, chlorogenic and ferulic acid were identified (Kitanov, 1992). Two essential triterpenoid saponins, that is, gypsogenin and oleanolic acid were isolated from the *S. media* (Figure 2) [34]. Three novel metabolites; 2, 4, 5, 7 tetramethyloctane, 2,2,4-trimethyloctan-3-one, 6-methyl heptyl-3′-hydroxy-2′ methylpropanoate were isolated from aerial parts of *S. media*. These metabolites displayed exceptional anti-obesity and anti-inflammatory activities (Figure 3) [35].

Important dietary supplements were identified in leaves extract of *S. media* through GC-MS technique. The metabolites are methyl stearate, hexacosanyl palmitate, ß-sitosterol, 6,7-dimethyl heptacosane, 3-methyl-6- hydroxy-heneicos-3-enyl acetate, triacontanoic acid, tricontanol, hentriacontane and pentacosanol [36]. Fresh and mature leaves are appraised to contain high proportion of ascorbic acid, carotenoids and tocopherol [37]. The leaves is also appraised to contain important essential and non-essential amino acids such as glycine, alanine, lysine, thymine, uracil, aspartic acid, glutamic acid, serine, proline, thymidine, tyrosine, 2- histidine, g-aminobutyric acid and threonine [38]. Aside amino acid, mature leaves and stem of *S. media* was reported to contain important flavonoids such as parietin, questin, daucosterol, anthraquinones emodin, 1-hexacosanol, kaempferol-3,7-ß-L-dirhamnoside and stellariose [13, 39, 40]. Phytochemical investigation of leaf extract of *S. media* revealed the presence of high proportion of total saponins (1.19 μg/g), total phenolic (45.5 ± 0.25 mg/g) and total flavonoid (1.4 mg/g) [41, 42]. Essential dietary minerals identified in *S. media* nickel, zinc, copper, sodium, cobalt, magnesium, lead, iron, manganese, cadmium and mercury [41].

### 2.5 Pharmacological activities of *Stelleria media*

Pharmacological appraisals of medicinal herbs are connected to the bioactive metabolites present [2]. Pharmacological assessments of different parts of *S. media* authenticated its anti-inflammatory, antioxidant, antimicrobial, anti-obesity, antidiabetic, anxiolytic and antileishmanial activities (Table 2). The pronounced pharmacological activities of *S. media* could be linked to the ethnopharmacological beliefs [42].

**Table 2:** Pharmacological activities of *S*. *media*.

*S. media* constituent	Experimental models	Pharmacological activities	References
Aqueous leaf extract of *S. media* with *Chenopodium album, Eclipta prostrata, Euphorbia pulcherima and Oxalis corniculata*	*In vitro*	Antifungal activities were examined against the growth and sporulation of fungus *Paecilomyces lilacinus*	[81]
Alkaloids and phenolics of whole plant extracts of *S. media*	*In vitro*	Antibacterial activities were examined on clinical isolates *of E. coli, S. typhi, K. pneumonia, Staph. aureus, Pseudomonas* *Aeruginosa* and *Bacillus subtilis*	[42]
Methanolic leaf extract of *S. media* (L.) Vill	*In vivo* (mice)	The anti-inflammatory and analgesic effect was studied using albumen induced paw oedema and formalin-induced paw lick	[30]
Lyophilized juice (LJ) of *S. media*	*In vitro* and *in vivo* (mice)	Anti-obesity activity was assessed by evaluating the inhibitory activity of LJ on pancreatic amylase and lipase and measurement of plasma triacylglycerol levels after oral administration of lipid emulsion	[41]
Ethanol extract *S. media*	*In vitro* and i*n vivo* (mice)	The hyperglycemic and hyperlipidemic effects was assessed and show decrease in fasting blood level	[48]
Crude extract of *S. media*	*In vivo* (Sprague-Dawley) rats	Androgenic and antioxidant activity was investigated the effects of ameliorating vital organ damage and spermatogenesis impairment induced by Dichlorvos following sub-chronic exposure	[12]
Methanolic extracts of leaf of *S. media*, *Cajanus cajan* and root of *Tetracera potatoria*	*In vitro*	Pronounced anti-oxidant activities was observed by DPPH radical scavenging and FRAP assays	[32]
Stellarmedin A isolated from *S. media*	*In vitro*	Antiproliferative and peroxidase activities from *S. media.* Stellarmedin A affects the initial stage of HSV-2 infection and inhibit the proliferation of promyelocytic leukemia HL-60 and colon carcinoma LoVo cells	[31]
Methanolic extract of *S. media*	*In vivo* (Wistar rats)	Methanolic extract show a strong anti-obesity effect with LD50 found to be more than 5000 mg/kg.	[16]
Sub-fraction EAF5 of bioactive ethyl acetate fraction derived from the methanol extract	*In vivo* (mice)	The anxiolytic activity show pronounced activities at dose of 20 mg/kg, po	[23]
Methanolic extract of *S. media*	*In vitro*	Antioxidant activity of 1020 ± 0.68 μg/ml	[37]
Aqueous and ethanolic extracts of chickweed herb	*In vitro*	The aqueous extract decreased intracellular ROS production by fibroblasts in a concentration-dependent manner and also reduced intracellular ROS production	[41]

### 2.6 Anti-inflammatory activities

Few studies appraised the anti-inflammatory potential of *S. media* in animals. Till date, only the *in vitro* appraisal of crude or purified fractions was reported. The inflammatory effect of methanolic leaf extract (MLE) was investigated on test rats of different body weights (100 mg/kg, 300 mg/kg and 500 mg/kg). Pronounced reduction in inflammation was exhibited on formalin-induced paw lick and albumen induced paw oedema after oral administration of MLE, indomethacin (5 mg/kg b.w) and distilled water (10 mg/kg b.w). Also, MLE considerably inhibited egg albumen-induced paw oedema at p < 0.05 [30].

The inhibition of hyaluronidase, lipoxidase and collagenase potentials of aqueous and ethanolic extracts at concentrations of 50–500 μg/mL, 10–200 μg/mL (hydrogen peroxide, H_2_O_2_ and O_2_^-^, superoxide anion), 5–50 μg/mL (NO^·^, nitrogen oxide) and 100–500 μg/mL (ONOO^−^, peroxynitrite) was appraised *in vitro* in cell-free systems via high-performance liquid chromatography coupled with Diode Array Detector and Ion Trap Mass Detector. Ethanolic extract exhibited significant scavenging activity against the radicals with scavenging concentration at 50% of 132.8 ± 3.9 μg/mL (H_2_O_2_), 16.5 ± 0.4 μg/mL (NO^·^) and 11.9 ± 1.1 μg/mL (ONOO^−^). However, aqueous extract displayed significant inhibitory concentration against superoxide anion (62.7 ± 8.1 μg/mL) when compared with ethanolic extract. Apigenin glycoside was appraised as bioactive metabolite in the extracts [43].

### 2.7 Antioxidant activity

The scavenging potential of *S. media* is appraised using 2,2-diphenyl-1-picrylhydrazyl (DPPH) and ferrulic reducing antioxidant potential (FRAP). The scavenging activity of apigenin glycosides in aqueous extract of *S. media* leaf is appraised on human skin keratinocytes and fibroblasts after ultra violet irradiation via DPPH assay. The extract displayed significant inhibitory activity of 62.75 g/mL against xanthine-xanthine oxidase system. The activity is linked to the decrease in the production of intracellular ROS exhibited by the flavonoid-rich extract [44]. In a similar study, alcoholic leaf extract of *S. media* is appraised in Sprague-Dawley rats (25 mg/kg/bw/day) for their potential in ameliorating spermatogenesis impairment and vital organ damage stimulated by Dichlorvos. The leaf extract significantly ameliorate epididymal (29.91%), liver (20.16%), body (10.73%) and testes (32.21%) weight which causes severe mutilation to reproductive organs and liver hepatocytes [12]. The phytochemical appraisal of leaf extract of *S. media* revealed the presence of tannins, phlobatannins and flavonoid. The crude extract exhibited strong free radical scavenging activity of 76% and 79% via DPPH and FRAP respectively [32].

### 2.8 Antimicrobial activity

The effect of *S. media* crude extract or purified fractions on microbial infections has been comprehensively studied. The first antibacterial activity of *S. media* was appraised on phenolic and alkaloids-rich aqueous and chloroform leaf extracts with concentrations of 62.5, 125, 250 and 500 mg/ml. The extracts significantly inhibit the microbial growth of *S. aureus, E. coli, S. typhi, P. aureginosa, K. pneumonia* and *B.cereus* [42]. The aqueous, methanol and ethanol leaf extracts of *S. media* were appraised on *E. coli, S. epidermidis, S. pyogenes,S. typhimurium, E. cloacea, P.vulgaris, S. aureus, K. pneumoniae, S. marcescens* and *P. aeruginosa.* The extracts significantly inhibited the microbial growth of the gram positive and negative bacteria [45]. In a recent study, peptides genepro-SmAMP2 and β-actin gene isolated from *S. media* significantly displayed strong inhibitory activity against the tested bacterial isolates whereas Sm-AMP-X exhibited significant inhibitory activity against phytopathogenic fungi due to the N- and C- terminal regions of the specified peptide [46, 47].

### 2.9 Anti-obesity activity

The anti-obesity potential of ethanolic and methanolic leaves and stem crude extracts was appraised in induced female Wistar mice. The phytochemical appraisal of methanolic leaf extract revealed the presence of flavonoids and β-sitosterol. The alcoholic leaf extract significantly inhibit growth of calorie in the test organisms with lethal dosage at 50% (LD_50_) of body weight less than 5000 mg/kg [16]. The pancreatic lipase potential of lyophilized juice (LJ) was investigated on plasma triacylglycerol in Swiss albino mice at dosage rate (400–900 mg/kg) for 42 days. LJ significantly reduced lipase and pancreatic amylase growth rate whereas triacylglycerol level is increased. Similarly, body and liver weight, retropertoneal adipose tissue levels were considerably suppressed which led to noticeable decrease in total triglyceride and cholesterol (900 mg/kg b.w) [41].

### 2.10 Antidiabetic activity

The antidiabetic appraisal of alcoholic leaf extract of *S. media* was assessed for their hyperlipidemic and hyperglycemic potential in alloxan induced diabetic mice using established documented procedures. Significant inhibitory activity was exhibited by pancreatic β-glucosidase and α-amylase. This instigated significant reduction in fasting blood sugar, serum transaminase and HbA1c (−48.4%) of tested organism when compared to control model. Also, blood glucose levels were acceptably maintained [48]. In a similar study, flavonoids, terpernoids, glutamic acid and arginine appraised in alcoholic leaf extract displayed significant antidiabetic effect on alloxan induced diabetic mice [49].

### 2.11 Anxiolytic activity

The tranquilizing and ability to lessen anxiety of methanol, aqueous, petroleum ether and chloroform extracts of *S. media* leaves at body weights of 50, 100, 200 and 400 mg/kg was appraised in mice via EPM model, diazepam (standard drug) (2 mg/kg) by actophotometre. Methanolic extract and diazepam displayed comparable activities in lengthening the time spent by test organisms in open arms and improve locomotory behaviour [23]. Ethyl acetate fraction of bioassay guided isolation of methanolic extract of *S. media* was assessed on Swiss albino mice via EPM model. The fraction and diazepam exhibited comparable activity at 20 mg/kg, po and 2 mg/kg respectively, thus, justifying the ethnomedical claim of *S. media* in alleviating anxiety [23].

### 2.12 Antileishmanial activity

Up to date, few studies have appraised the immunology or inhibitory effect of *S. media* against Leishmania parasites. In recent times, alcoholic extracts of *S. media* was appraised on *Leishmania tropica* KWH23 promastigote isolated from an infected patient in Peshawar, Pakistan, sprouted in M199 medium with HEPES buffer, 10% FCS, penicillin and streptomycin. The isolated parasite was cultured on a microtitter plate and incubated at 24 °C for 72 h. The inhibitory effect of the alcoholic extracts was appraised in microscope with aid of Neubauer counting chamber. From observation, methanolic and ethyl acetate extracts significantly inhibited the growth of *L. tropica* with inhibitory activity of 185.9 ± 7.5 *μ*g/mL and 36.4 ± 2.5 *μ*g/mL respectively, however, glucantime exhibited weak activity of 5.6 ± 0.25 *μ*g/mL [14].

### 2.13 Toxicological profile

Herbal drugs is viewed as the safest and innocuous therapeutic system, however, recent side effects reported from the use of herbs has significantly disrupted its safety or efficacy claims and also, most herbal plants are not well cited nor documented [50]. The toxicological appraisal of herbal drugs will facilitates and justifies its authenticity and safety. However, few toxicological reports on *in vitro* and *in vivo* applications of different parts of *S. media* raise questions on the usage of the plant parts. However, the toxicological and immune-stimulatory effect of *S. media* was appraised on Swiss mice by critical assessment of serum biochemistry and haematology of the test organisms. The assessment was based on discrepancy in mean corpuscular volume, packed cell volume and white blood cell whereas albumin, creatinine, total protein and serum bilirubin levels were assessed on the kidney and liver. The extracts significantly increase the lymphocyte counts (4.6 – 8.6 × 10^3^/mm^3^), however, 4.3 × 10^3^/mm^3^ increment was observed in the control mice at P < 0.05. The extracts exhibited significant reduction in protein level of 3.3–3.8 g/dl and insignificant immune-stimulatory effect was exhibited on the haematology [32]. Phytochemical appraisals of different parts of *S. media* revealed the presence of important metabolites such as saponins, cardiac glycosides, phenolics, terpenoids, flavonoids, phenolics, 1, 8-cineole, linalool, and mentol. These metabolites at high levels could cause contact dermatitis, diarrhoea, cyanosis, nausea, dizziness and erythema multiforme [51, 52]. In addition, nitrates identified in *S. media* are presumed to cause vertigo, weakness, headache, difficulty in breathing, cutaneous staining on fingers or lips and gestational pain [51, 52].

## 3 Discussion

Medicinal plants are referred to as major source of nourishments and natural metabolites for maintaining sound health [53]. They are also referred to as indispensable natural compounds with vast number of pharmacological activities [54, 55, 56]. Since inception, medicinal plants and herbal drugs are used in treatment of various kinds of acute and chronic diseases [2, 56, 57]. The therapeutic or curative significance of *S. media* in has gained global recognition mostly because of its relevance in traditional medicine. These curative applications could be due to bioactive metabolites such as minerals, vitamins, and other essential nutrients appraised [34]. Phytochemical assessment and dietary appraisals of different parts of *S. media* revealed presence of essential antioxidant vitamins, phenolic compounds and glycosides [12, 13, 23, 34, 41].

Generally, inflammation is appraised as body reaction to oxidative stress or pathogenic invasion and is often associated with severe pain or swelling of body surface and could lead acute or chronic diseases such as rheumatoid arthritis, cystic fibrosis, osteoarthritis, allergies, and cancer [58, 59]. Plant secondary metabolites such as flavonoid glycosides and aglycone, g-aminobutyric acid, glutamic acid, anthraquinones emodin, and aspartic acid identified in *S. media* is reported to exhibit significant antimicrobial, immunomodulatory and anti-inflammatory effects in human cells, animal models and pathogenic microbes [13, 32, 33, 38]. In addition, anthraquinones emodin, flavonoid glycosides and aglycone demonstrated pronounced therapeutic effects on inflammatory bowel syndrome and significantly inhibit related adverse conditions such as gastrointestinal pain and bloody diarrhoea [60]. Also, tannin-rich *S. media* extract exhibited strong prophylaxis potential and also displayed significant antimalarial activity [61].

Different parts of chicken weed are significantly used as therapeutic substances in treatment of inflammation, mental health disorder and tension [23, 62, 63]. Phytochemical appraisals of flower extract of *S. media* revealed presence of some secondary metabolites with great potential in activating specified genes which dictate its therapeutic potential such as antimicrobial and anti-protozoal [47, 63]. Certain plant metabolites such as tannins and saponins contributed to pronounced inhibitory activities displayed by *S. media* leaf against different kinds of dermal infection caused by *Staphylococcus aureus*, however, alkaloids exhibited strong inhibitory activity against bacterial infections by *P. aeruginosa and S. aureus* [64].

Diabetes is one of the communal diseases of man contributing to stern socio-cultural, economic and health influences in humid, middle-income, sub-Saharan Africa, Southeast Asia and South America [65]. It is associated with metabolic disorders relating to endocrine abnormalities, inflammation and oxidative stress [65]. The ability of *S. media* leaf to lessen endocrine abnormalities in animals is related to the scavenging activity and inhibitory effects of glycosidase and amylase on enzymes which contributed to carbohydrate metabolism [66, 67]. In addition, metabolites such as tannins, saponins, flavonoids and flavonoid glycosides contribute to antidiabetic potential of chicken weed. Tannins is appraised to regulate blood glucose level and lipid profile concentration without increasing adiposity [68]. Saponins contributed to management of serum glucose level in diabetic patients [69, 70]. Flavonoids undergo metabolic processes to combat diabetic complications and enhance insulin secretion, proliferation of pancreatic β-cells. It also reduces oxidative stress, insulin resistance, apoptosis and inflammation in muscles [71, 72].

Antiobesity is a global health concern, majorly caused by nutrition and food supplements. The imprudent intake of fatty food substances has been appraised to significantly lead to more than 70% of global obesity. In recent times, obesity is linked to most cases of diabetes, hypertension, cancer and high blood pressure [73]. Flavonoids and *β-*sitosterol from *S. media* significantly inhibit lipase and pancreatic amylase growth rate in Swiss albino mice while decreasing total triglyceride and cholesterol [41]. Herbs with potential in healing wound and incision has been in search ever since inception of man [74]. The wound healing and anti-inflammatory potentials of medicinal plant is appraised to depend on tannins content [75, 76]. The wound healing potential of *S. media* is appraised to be due to presence of alkaloids [75, 77]. The production of free radicals in man has been identified as health challenge contributing to several neurological syndrome, cancer, endocrine illness and aging [78]. Phenolic and flavonoids is appraised to significantly promote collagen synthesis and cross-linking of collagen, however, it is discovered to abridge inflammation period [79]. Saponin is effective in management of inflammation, wound, bleeding, endocrine illness and neurological syndrome [75, 80].

## 4 Conclusion and perceptions

An extensive literature assessment on *S. media* revealed assortment of bioactive metabolites. In Traditional medicine, *S. media* has been used in treatment of obesity, diabetes, dermal infections, inflammation, gastric ulcers and stomach cramps. Major bioactive metabolites such as alkaloids, tannins, phenolic compounds, phenolic acids, saponins, triterpenoids, flavonoids and flavonoids aglycone are appraised to contribute to strong therapeutic potency. Crude extracts and isolated pure compounds from *S. media* displayed pronounced pharmacological activities justifying the different ethnopharmacological and ethnobotanical applications of *S. media*. Researchers have done little in authenticating the therapeutic properties displayed by this plant. However, further appraisals to isolate active bioactive metabolites responsible for the marked pharmacological activities could lead to development of novel therapeutic compounds.

## Declarations

### Author contribution statement

O. Oladeji and A.K. Oyebamiji: Conceived and designed the experiments; Performed the experiments; Analyzed and interpreted the data; Contributed reagents, materials, analysis tools or data; Wrote the paper.

### Funding statement

This research did not receive any specific grant from funding agencies in the public, commercial, or not-for-profit sectors.

### Competing interest statement

The authors declare no conflict of interest.

### Additional information

No additional information is available for this paper.
